# Plasma cell-rich related acute rejection in kidney transplant: A case report and review of the literature

**DOI:** 10.1097/MD.0000000000030493

**Published:** 2022-09-09

**Authors:** Yao-Yu Tsai, Lee-Moay Lim, Hung-Tien Kuo, Yi-Chun Tsai

**Affiliations:** a Department of Internal Medicine, Kaohsiung Medical University Hospital, Kaohsiung Medical University, Kaohsiung, Taiwan; b School of Medicine, College of Medicine, Kaohsiung Medical University, Kaohsiung, Taiwan; c Division of Nephrology, Department of Internal Medicine, Kaohsiung Medical University Hospital, Kaohsiung Medical University, Kaohsiung, Taiwan; d Division of General Medicine, Department of Internal Medicine, Kaohsiung Medical University Hospital, Kaohsiung Medical University, Kaohsiung, Taiwan.

**Keywords:** acute rejection, kidney transplant, plasma cell

## Abstract

Plasma cell-rich acute rejection (PCAR), a subtype of T cell-mediated rejection, is a relatively rare type of acute allograft rejection, that is usually associated with a higher rate of graft failure. However, it is difficult to diagnose PCAR precisely.

**Patient concerns:**

A 45-year-old woman who had received a kidney transplant presented with acute kidney injury and uremic symptoms approximately 1 year after transplantation.

**Diagnosis:**

A renal biopsy was performed and pathological examination revealed marked inflammation with abundant plasma cells in areas within interstitial fibrosis and tubular atrophy. The patient was diagnosed with PCAR and chronic active T cell-mediated rejection (CA-TCMR) grade IA.

**Interventions:**

Immunosuppressants were administered as tacrolimus (2 mg twice daily), mycophenolate mofetil (250 mg twice daily), and prednisolone (15 mg/day) for suspected PCAR.

**Outcomes:**

The patients showed rapid deterioration in kidney function and reached impending graft failure.

**Lessons:**

PCAR is often associated with poor graft outcome. The high variability in tacrolimus levels could contribute to poor patient outcomes, leaving aggressive immunosuppressive therapy as the remaining choice for PCAR treatment.

## 1. Introduction

Plasma cell-rich acute rejection (PCAR) is a relatively rare type of acute allograft rejection, characterized by the infiltration of plasma cells into the renal parenchyma. The frequency of PCAR among allograft rejections had varied from 2% to 14%.^[1]^ PCAR is mainly defined as the number of plasma cells >10% of graft infiltrating cells,^[2]^ with approximately 40% to 60% of PCAR resulting in graft failure within a year. Risk factors for PCAR include a longer duration from transplantation to onset,^[3]^ poor medication adherence, and tapering doses of immunosuppressants.^[4]^ Additionally, plasma cell-rich histological features might be associated with drug reactions and infections such as post-transplant lymphoproliferative disorder (PTLD) nephropathy, reflux nephropathy and BK virus infection.^[5,6]^

PCAR is often categorized into subtypes of T cell mediated rejection (TCMR) under the Banff criteria,^[7]^ although PCAR and antibody-mediated rejection may share similar characteristics. Hasegawa et al found 9 of 40 patients with PCAR were categorized as having antibody-mediated rejection based on the 2013 Banff criteria.^[6]^ Hamada et al^[8]^ reported that among 6 patients with PCAR, 2 also had antibody-mediated rejection with donor-specific antibodies. On the 2017 Banff meeting, new criteria for chronically active T cell-mediated rejection (CA-TCMR) was defined using the inflammation score of areas with interstitial fibrosis and tubular atrophy (*i-IFTA*).^[7]^ Nevertheless, case reports of PCAR that fulfilled the new CA-TCMR criteria are still limited.

In this report, we discuss the pathological features and clinical course of a rare case of PCAR.

## 2. Case presentation

A 45-year-old female patient with hypertension and dyslipidemia developed end stage renal disease after pre-eclampsia, and had received continuous ambulatory peritoneal dialysis since 2010. She underwent a kidney transplantation in December 2019 from a 48-year-old cadaver donor. The pre-transplantation panel reactive antibody test showed major histocompatibility complex class I 1.3% and major histocompatibility complex II 0.4%. Her serum creatinine level was 11.57 mg/dL (estimated glomerular filtration rate (eGFR): 3.59 mL/min/1.73 m^2^) before transplantation. After the transplantation, the patient received induction immunosuppressive therapy with basiliximab and methylprednisolone. She received maintenance anti-rejection therapy with prednisolone (10 mg/day), mycophenolate mofetil (MMF) (500 mg twice daily), and tacrolimus (2.5 mg twice daily). Her kidney function improved (eGFR: 83.06 mL/min/1.73 m^2^) after transplantation and induction therapy. Owing to stable renal function, prednisolone was gradually discontinued in March 2020. The tacrolimus dosage was tapered to 4 mg/day in September 2020, and that of MMF was tapered to 250 mg twice daily in October 2020. The patient also received prophylactic antimicrobial medication with valganciclovir, sulfamethoxazole and trimethoprim for half-a-year after transplantation.

However, her serum creatinine became elevated to 1.05 mg/dL (eGFR: 56.93 Ml/min/1.73 m^2^) while FK506 level rose to 11.3 μg/L in December 2020. Acute kidney injury (AKI) induced by calcineurin inhibitor toxicity was suspected. After adjusting the dosage of immunosuppressant, deterioration of the kidney function was observed. The patient was hospitalized for further survey of AKI in January 2021. Proteinuria (urine protein-creatinine ratio: 0.74 g/g) and hypercalcemia with tertiary hyperparathyroidism were also observed. Apart from the slightly elevated immunoglobulin A levels, serum immunological tests revealed no abnormal findings. Renal sonography revealed a normal size and contour of the transplanted kidney.

**Figure 1.**
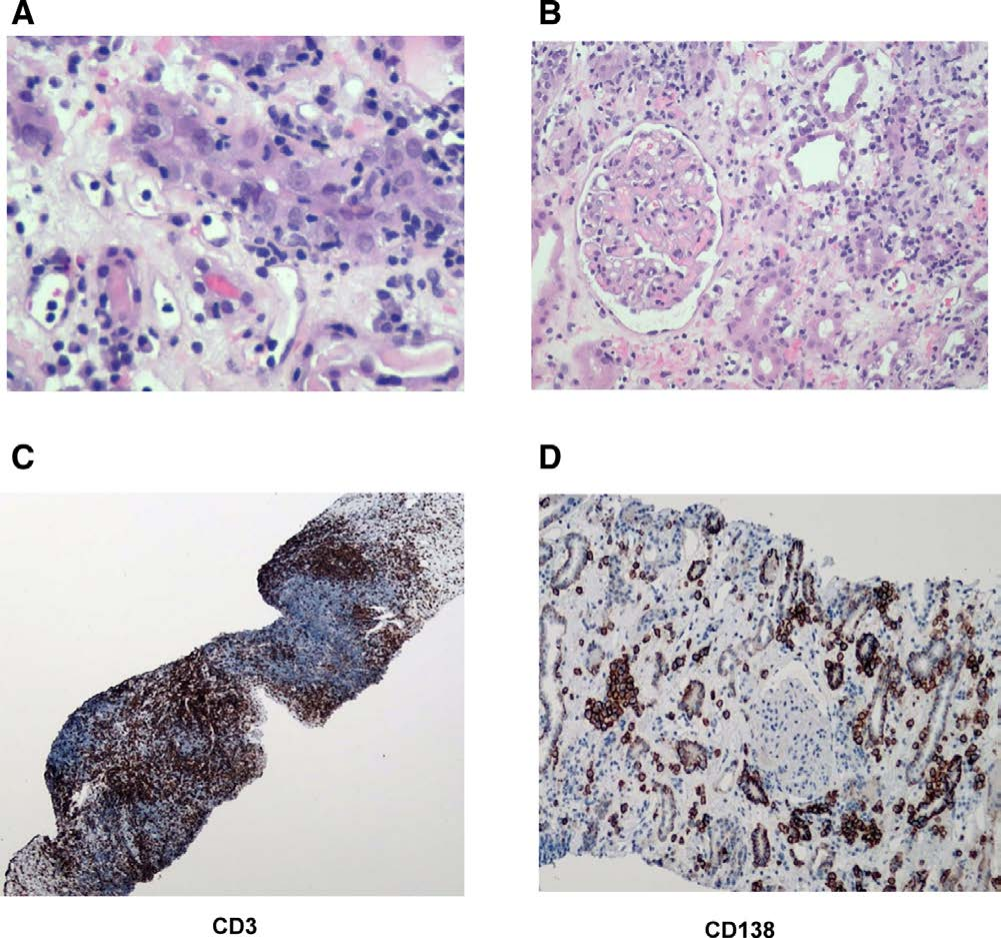
Immunohistochemistry. (A) Infiltration with plasma cell and monocyte examined using hematoxylin and eosin H&E staining (400×). (B) Moderate tubulitis lesion detected by H&E staining (200×). (C) T-lymphocytes infiltration in interstitium, tubules and glomeruli using CD3 staining (brown, 50×). (D) plasma cell scattered in tubules and peritubular area detected by CD138 staining (brown, 100×). CD = cluster of differentiation, H&E = hematoxylin and eosin.

Renal biopsy was performed and revealed interstitial fibrosis in 35% of the cortical area (2) and moderate chronic inflammatory infiltration in 40% of the non-scarred cortex (2). Discernible inflammation with plasma cells infiltration in 60% of the scarred area (*i-IFTA*3) is shown in Figure 1A. Tubular atrophy was detected in 35% of area (2), and moderate tubulitis (2) was observed in non-scarred or scarred areas with moderate inflammatory cells in the luminal space of peritubular space (2). Neither vasculitis nor glomerulitis were observed (Fig. 1B). Immunohistochemical staining revealed scattered T lymphocytes in the interstitial space, glomeruli and renal tubules with cluster of differentiation (CD)3 staining (Fig. 1C). Scattered macrophages were observed throughout the interstitium and glomeruli by CD68 staining. Plenty of plasma cells (>10% of graft infiltrating cells) were identified throughout the renal tissue using CD138 staining (Fig. 1D). Cytomegalovirus or BK virus was not detected in the blood or kidney, and negative C4d immunoreactivity was observed. Immunofluorescence staining revealed no immune-complex deposition in the glomeruli. Finally, the patient was diagnosed with CA-TCMR, grade IA (*ti*2, *i-IFTA*3, *t*2) (Table 1). Plasma cell-rich rejection was also considered because of the abundance of plasma cells in the inflamed interstitium.

**Table 1:** Histological diagnosis of the case according to Banff lesion score.

Banff lesion	Abbreviation	Score	Description
Interstitial inflammation	*i*	2	26%–50%
**Tubulitis**	***t***	**2**	**5–10/tubular cross section**
Intimal arteritis	*v*	0	None
Glomerulitis	*g*	0	None
Peritubular capillaritis	*ptc*	2	≥1 leukocyte in ≥10% of ptcs with max. of 5–10/ptc
Complement 4d	*C4d*	0	None
Interstitial fibrosis	*ci*	2	26%–50%
Tubular atrophy	*ct*	2	26%–50%
Vascular fibrosis Intimal thickening	*cv*	0	None
GBM double contours	*cg*	0	None
Mesangial matrix expansion	*mm*	0	None
Arteriolar hyalinosis	*ah*	0	None
Hyaline arteriolar thickening	*aah*	0	None
**Total inflammation**	***ti***	**2**	**26%–50%**
**Inflammation in the area of interstitial fibrosis and tubular atrophy**	***i-IFTA***	**3**	**>50%**

**Figure 2.**
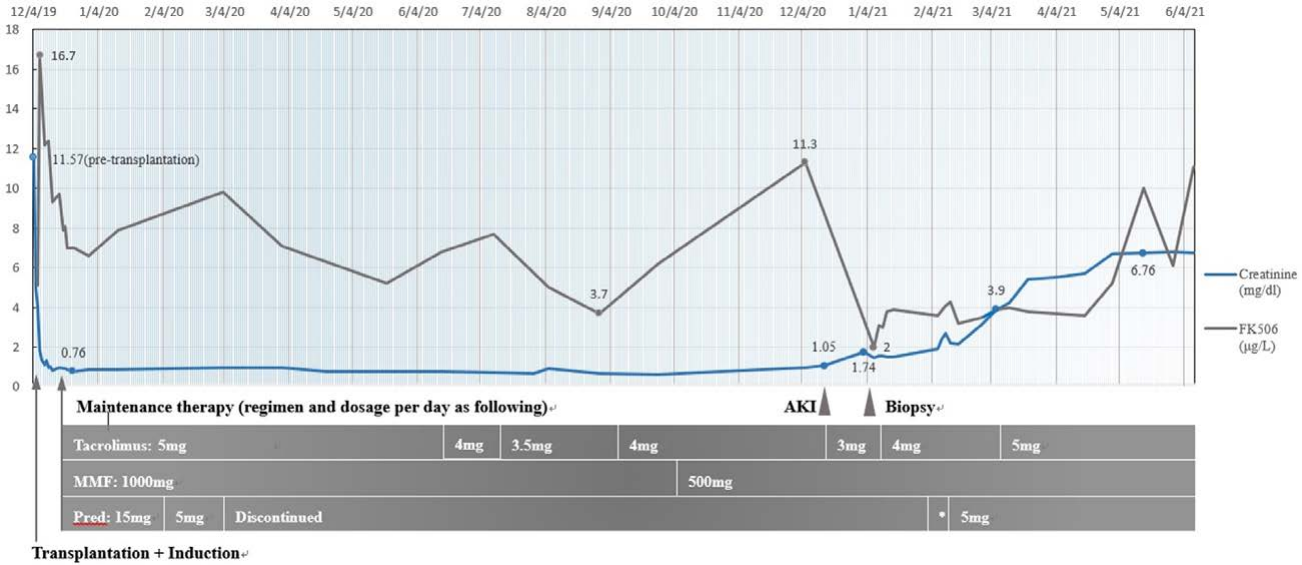
Summary of clinical course with treatment regimen, serum creatinine, and FK506 trough level in this patient. *: The patient had 2 weeks use of 15 mg/d prednisolone after PCAR and CA-TCMR was diagnosed. CA-TCMR = chronic active T cell-mediated rejection, PCAR = plasma cell-rich acute rejection, Pred = prednisolone.

In AKI patients with CA-TCMR, immunosuppressive medications were escalated to tacrolimus 2 mg twice daily, MMF 250 mg twice daily, and prednisolone 15 mg/day; however, the patient could not tolerate the side effects of steroids. Progressive deterioration in kidney function (eGFR: 6.61 mL/min/1.73 m^2^) and uremic symptoms, such as edema and oliguria, were noticed after adjusting medication (tacrolimus to 2.5 mg twice daily, MMF 250 mg twice daily and prednisolone 5 mg daily) (Fig. 2). Thus, graft failure was suspected and arteriovenous graft creation for hemodialysis was suggested.

## 3. Discussion and Conclusions

This report describes a case of PCAR that also fit the diagnosis of CA-TCMR. Since multiple factors such as infection, drug hyper-reaction, PTLD nephropathy and reflux nephropathy might result in plasma cell-rich features,^[6]^ clinical physicians should exclude these confounding factors. In this case, serological testing and immunohistochemistry staining did not detect cytomegalovirus or BK viruses, whereas pathology sampling lacked typical histological findings of PTLD. Accordingly, the patient was diagnosed with CA-TCMR and classified as having PCAR.

Accumulating evidence suggests that medical adherence and a reduced dosage of immunosuppressant may be risk factors for PCAR.^[9]^ Lerut et al^[10]^ found a trend of increased plasma cell infiltration in patients with poor medical compliance compared with those with good medical compliance. Another study indicated that non-adherence to immunosuppressive medications is a major cause of B cell-mediated acute rejection.^[9]^ Our case had poor compliance with immunosuppressants and the dosage of medication was changed during the treatment period. This may be one reason for the occurrence of PCAR.

In addition, maintenance and monitoring of immunosuppressive drug levels are essential for kidney transplantation. Among the immunosuppressive regimens, calcineurin inhibitors are extremely effective; however, calcineurin inhibitor overdose is associated with nephrotoxicity and opportunistic infections. It is also crucial to monitor tacrolimus trough levels because of the narrow window of effectiveness. Kidney Disease: Improving Global Outcomes (KDIGO) guidelines suggest that the appropriate tacrolimus trough levels in the early post-transplant stage is 5 to 15 ng/mL.^[11]^ A previous study found that patients with tacrolimus trough levels between 5.35 and 7.15 ng/mL had a lower acute rejection rate than those with tacrolimus trough level <5.35 ng/mL and had a lower infection rate than those with tacrolimus trough level >7.15 ng/mL.^[12]^ The variability in tacrolimus trough levels is also an important risk factor for graft rejection. Huang et al reported that the percent coefficient of variation of tacrolimus trough level within 6 months in patients with acute rejection was significantly higher than that in patients without acute rejection.^[13]^ Moreover, the high variability of tacrolimus trough levels might be related to poor drug adherence and drug-drug interactions. Wu et al reported that medication adherence could be improved by altering the dosage from twice a day to once a day in stable patients, thereby lowering the variability in tacrolimus trough concentration.^[14]^ In our case, the patient’s coefficient of variation percentage of tacrolimus trough level within 6 months was 34.9%, which might contribute to the higher rejection rate. Additionally, the twice-a-day tacrolimus regimen might induce poor compliance, resulting in high variability in tacrolimus trough levels.

To date, there is no gold standard for efficient treatment of PCAR. Abbas et al reported that they treated with PCAR patients with methylprednisolone (500 mg/kg), 7 sessions of plasmapheresis, antithymocyte globulin (3–5 mg/kg/day for 10 days), rituximab (2 doses at 375 mg/m^2^), and bortezomib (1 cycle at 1.3 mg/m^2^), with 2-year graft survival rate after rejection of 90%.^[15]^ Other aggressive salvage therapies include intravenous immunoglobulin^[4]^ and steroid pulse therapy.^[16]^ However, evidence for the most effective treatment for PCAR remains limited. Further studies are necessary to develop more efficient therapeutic strategies for treating PCAR.

In conclusion, we report a rare case of PCAR combined with CA-TCMR and explore the reasons for treatment failure. Poor medical compliance and high variability in tacrolimus trough level may promote the onset and progression of graft rejection.

## Author contributions

**Conceptualization:** Lee-Moay Lim, Hung-Tien Kuo, Yi-Chun Tsai.

Data curation: Yao-Yu Tsai.

Methodology: Yao-Yu Tsai, Yi-Chun Tsai.

Resources: Lee-Moay Lim, Hung-Tien Kuo, Yi-Chun Tsai.

Supervision: Yi-Chun Tsai.

Validation: Yi-Chun Tsai.

Writing – original draft: Yao-Yu Tsai.

Writing – review& editing: Yi-Chun Tsai.

## Acknowledgments

The authors would like to thank the Department of Pathology, Kaohsiung Medical University Hospital, Kaohsiung, Taiwan.
